# Development and multi-center cross-setting validation of an explainable prediction model for sarcopenic obesity: a machine learning approach based on readily available clinical features

**DOI:** 10.1007/s40520-025-02975-z

**Published:** 2025-03-01

**Authors:** Rongna Lian, Huiyu Tang, Zecong Chen, Xiaoyan Chen, Shuyue Luo, Wenhua Jiang, Jiaojiao Jiang, Ming Yang

**Affiliations:** 1 Center of Gerontology and Geriatrics, West China Hospital, Sichuan University, Chengdu, China; 2 Department of Geriatric, Zigong Affiliated Hospital of Southwest Medical University, Zigong, China; 3 Rehabilitation Center, West China Hospital, Sichuan University, Chengdu, China; 4 National Clinical Research Center for Geriatrics, West China Hospital, Sichuan University, Chengdu, China

**Keywords:** Obese sarcopenia, Machine learning, Prediction model, Diagnostic performance

## Abstract

**Objectives:**

Sarcopenic obesity (SO), characterized by the coexistence of obesity and sarcopenia, is an increasingly prevalent condition in aging populations, associated with numerous adverse health outcomes. We aimed to identify and validate an explainable prediction model of SO using easily available clinical characteristics.

**Setting and participants:**

A preliminary cohort of 1,431 participants from three community regions in Ziyang city, China, was used for model development and internal validation. For external validation, we utilized data from 832 residents of multi-center nursing homes.

**Measurements:**

The diagnosis of SO was based on the European Society for Clinical Nutrition and Metabolism (ESPEN) and the European Association for the Study of Obesity (EASO) criteria. Five machine learning models (support vector machine, logistic regression, random forest, light gradient boosting machine, and extreme gradient boosting) were used to predict SO. The performance of these models was assessed by the area under the receiver operating characteristic curve (AUC). The SHapley Additive exPlanations (SHAP) approach was used for model interpretation.

**Results:**

After feature reduction, an 8-feature model demonstrated good predictive ability. Among the five models tested, the support vector machine (SVM) model performed best in SO prediction in both internal (AUC = 0.862) and external (AUC = 0.785) validation sets. The eight key predictors identified were BMI, gender, neck circumference, waist circumference, thigh circumference, time to full tandem standing, time to five-times sit-to-stand, and age. SHAP analysis revealed BMI and gender as the most influential predictors. To facilitate the utilization of the SVM model in clinical setting, we developed a web application (https://svcpredictapp.streamlit.app/).

**Conclusions:**

We developed an explainable machine learning model to predict SO in aging community and nursing populations. This model offers a novel, accessible, and interpretable approach to SO prediction with potential to enhance early detection and intervention strategies. Further studies are warranted to validate our model in diverse populations and evaluate its impact on patient outcomes when integrated into comprehensive geriatric assessments.

**Supplementary Information:**

The online version contains supplementary material available at 10.1007/s40520-025-02975-z.

## Introduction

The global demographic landscape is undergoing a significant transformation, with a substantial increase in the aging population, particularly among adults aged 65 years and older [1]. Within this population, obesity has risen steadily. According to the National Health and Nutrition Examination Survey (NHANES), more than one-third of adults aged 65 years and older were diagnosed with obesity, representing a significant public health concern [2]. Meanwhile, the aging process is often accompanied by a progressive loss of muscle mass and function, a condition known as sarcopenia [3].

The concurrent presence of sarcopenia and obesity can lead to a condition known as sarcopenic obesity (SO). These two conditions may synergistically exacerbate each other, creating a vicious cycle of fat accumulation and muscle depletion through a sedentary lifestyle, reduced mobility, dependency, and disability [4, 5]. This complex interplay results in the clinical entity of SO, which presents unique challenges in diagnosis and management.

The prevalence of SO demonstrated significant variability across different diagnostic criteria and study populations. Our recent research revealed that even using the same diagnostic criteria, the prevalence of SO was 13.2% in community-dwelling older adults [6], while it reached 45.3% in nursing home residents [7]. The impact of SO extends beyond its prevalence, as evidenced by numerous studies demonstrating its role as a strong and independent risk factor for various adverse health outcomes. These include an increased risk of falls, fractures [8], disability [9], comorbidities [10], unfavorable quality of life [11], and mortality [12], underscoring the critical need for early diagnosis and intervention.

Recognizing the importance of standardized diagnostic criteria, the European Society for Clinical Nutrition and Metabolism (ESPEN) and the European Society Association for the Study of Obesity (EASO) [13] recently released the first comprehensive diagnostic procedures for SO. This protocol emphasizes a two-step approach, beginning with an assessment of skeletal muscle function, followed by an evaluation of body composition. The ESPEN/EASO group highlights the importance of body composition measurements in the diagnostic process, advocating for the use of advanced techniques when available.

Currently, the common devices used for measuring body composition include computed tomography (CT), magnetic resonance imaging (MRI), dual-energy X-ray absorptiometry (DXA), and bioelectrical impedance analysis (BIA). However, each of these methods has significant limitations [14]. CT and MRI, while highly accurate, are expensive and time-consuming; moreover, CT poses the additional risk of radiation exposure. DXA’s accuracy can be affected by body thickness and hydration status, while BIA is an indirect method to measure body composition based on a specific equation for different populations [15, 16]. Of particular concern is the limited accessibility of these devices for community-dwelling older adults and nursing home residents. This lack of access may lead to the underdiagnosis of SO in these vulnerable populations. Consequently, there is a pressing need for the development of diagnostic tools based on readily available clinical features to facilitate the early identification of SO.

Machine learning, a subset of artificial intelligence that focuses on the development of algorithms capable of improving automatically through experience, has shown promising results in various areas of clinical diagnosis in recent years [17]. Several studies have demonstrated the efficacy of machine learning in predicting complex medical conditions, such as acute kidney injury [18] and skeletal muscle loss [19], often exhibiting excellent performance metrics. However, the adoption of machine learning in clinical practice has been hampered by concerns about the “black box” nature of many models, which can make it difficult for clinicians to understand and trust the predictions [20]. To address the issue, recent advancements in the field of explainable artificial intelligence have led to the development of techniques such as the SHapley Additive exPlanations (SHAP) approach [21]. This method allows for the interpretation of machine learning models and the visualization of individual variable predictions, potentially increasing the acceptability of these models in clinical settings.

Given the significant health implications of SO and the limitations of current diagnostic methods, there is a clear need for innovative approaches to its identification. The development of low-cost, convenient, and rapid machine learning models for SO diagnosis represents a promising direction in addressing this need. In this context, our study aims to develop and validate machine learning models for the diagnosis of SO using readily available clinical characteristics in community-dwelling older adults. To enhance the generalizability and reliability of our findings, we conducted a rigorous external validation of these models in multi-center nursing home residents. Furthermore, to improve the interpretability and clinical utility of these models, we employed the SHAP method for model explanation.

## Methods

This study was conducted in accordance with the Declaration of Helsinki and approved by the Biomedical Ethics Review Committee of West China Hospital, Sichuan University (No. 2021 − 965). All participants provided written informed consent prior to their enrollment in the study. To ensure comprehensive and transparent reporting of our research, we adhered to the Consolidated Reporting of Machine Learning Studies (CREMLS) checklist [22].

### Study populations

#### Derivation cohort

We consecutively and prospectively recruited participants from five communities in Ziyang City, China, between February and May 2022. All adults aged 60 years and older were eligible for inclusion. Individuals with any of the following conditions were excluded: (1) presence of metallic implants (i.e., pacemakers, implantable cardioverter defibrillators, or dental implants); (2) acute illness (i.e., trauma, acute coronary syndrome, or acute infection); (3) history of mental disorder, major cognitive impairment, or delirium; (4) history of skeletal muscle diseases (i.e., myositis, progressive muscular dystrophy, or myasthenia gravis); (5) amputation or bone fracture within the past six months; (6) visible edema; (7) major surgery within three months before enrollment; (8) refusal to participate; and (9) with missing data. Of the 1,587 eligible individuals approached, 1,431 (90.2%) consented to participate and were enrolled in the preliminary cohort.

For model development and validation, the cohort was randomly divided using a stratified sampling method to maintain the same proportion of sarcopenic obesity in both subsets. 70% (*n* = 1,002) of the participants were allocated to the training set for model development, while the remaining 30% (*n* = 429) were reserved for internal validation.

#### External validation cohort

To assess the generalizability of our model, we prospectively recruited an external validation cohort, consisting of residents living in 15 nursing homes in Zigong City, China, between September 2021, and July 2022. These nursing homes were selected to represent diverse care levels and socioeconomic backgrounds. The inclusion and exclusion criteria were identical to the development and internal validation cohort. Data collection methods and measurements were consistent across all cohorts to ensure comparability. Of 956 eligible residents, 832 (87.0%) agreed to participate and were enrolled for external validation.

### Data collection

Data were collected through a comprehensive approach. Trained interviewers conducted face-to-face, one-on-one interviews to gather questionnaire data. Concurrently, certified nurses performed anthropometric measurements and body composition assessments. The initial dataset for each participant comprised 51 candidate clinical characteristics, categorized as follows: demographic information (5 variables), anthropometric measurements (3 variables), disease history (19 variables), physical performance tests (19 variables), and mental health (5 variables). The details of these variables and the corresponding measurement methods are presented in Supplementary Table 1.

### Definition of sarcopenic obesity

The diagnosis of SO was based on the ESPEN/EASO [13] criteria. This definition requires the co-existence of altered muscle function and altered body composition (including excess adiposity and low muscle mass). Muscle function was assessed using handgrip strength (HGS), measured with a digital dynamometer (EH101, Xiangshan Inc., Guangdong, China). The maximum value of three attempts with the dominant hand was recorded. Low muscle strength was defined as HGS < 28 kg for men and < 18 kg for women [23]. Body composition, including skeletal muscle mass (SMM) and fat mass (FM), was measured using a multi-frequency segmental BIA device (InBody 770, Biospace, Seoul, Korea).

The ESPEN/EASO group suggested the adjustment of SMM for body weight (SMM/W) to determine low muscle mass. In this study, “low muscle mass” was defined as SMM/W < 38.2% for men, and < 32.2% for women [24]. Furthermore, “obesity” was defined as FM percentage of body weight (FM%) > 20.21% for men, and > 31.71% for women [25]. These cut-offs were chosen as they were developed based on Asian populations and conform to the ESPEN/EASO consensus.

### Feature selection and processing

We employed a two-step approach for feature selection and processing. First, to mitigate multicollinearity, we calculated the variance inflation factor (VIF) for each feature [26]. Features with a VIF exceeding 10 were eliminated from the model. Subsequently, we applied the Least Absolute Shrinkage and Selection Operator (LASSO) regression with random sampling for further feature selection. This process resulted in a final set of 24 features, which were used to develop the prediction models.

### Machine learning models

We developed and compared five machine learning models for SO prediction: support vector machine (SVM), logistic regression (LR), random forest (RF), extreme gradient boosting (XGBoost), and light gradient boosting machine (LightGBM). To optimize model performance, we employed five-fold Grid Search cross-validation for hyperparameter tuning. This process involved systematically working through multiple combinations of parameter tunes, cross-validating as it goes to determine which tune gives the best performance.

### Model evaluation

Model performance was evaluated using several metrics: the area under the receiver operating characteristic curve (AUC), sensitivity, specificity, positive predictive value (PPV), and negative predictive value (NPV), accuracy, F1-score, calibration. We also provided calibration curves and conducted decision curve analysis. We conducted both internal and external validation. Internal validation utilized 30% of the initial cohort (*n* = 429), which was randomly set aside during the initial data split. External validation was performed using the independent nursing home cohort (*n* = 832) to assess the models’ generalizability to different populations and settings.

### Model interpretation

To enhance the interpretability of our machine learning models, we employed the SHAP approach. This method provides both global and local explanations of model predictions. We calculated SHAP values for each feature to quantify their respective contributions to the model’s predictions. We generated several visualization tools to aid in interpretation: SHAP summary plots to show the overall impact of features, SHAP scatter plots to illustrate the relationship between feature values and their impact on predictions, SHAP force plots to visualize the contribution of each feature to individual predictions, and SHAP waterfall plots to demonstrate how each feature pushes the model output from the base value to the final prediction [21].

### Web application development

To bridge the gap between research and clinical practice, we developed a user-friendly web application based on the final prediction model using the Streamlit Python framework. The application allows healthcare professionals to input patient data and receive real-time predictions of SO probability. The application also generates a force plot for each participant, providing a visual representation of how different features contribute to the prediction.

### Statistical analysis

We conducted statistical analyses using R software version 4.2.3 (R Foundation for Statistical Computing, Vienna, Austria) and Python version 3.11.9 (Python Software Foundation, Amsterdam, Netherlands). The distribution of continuous variables was assessed using histograms and the Shapiro-Wilk test. All continuous variables in this study exhibited normal distribution and were therefore presented as mean with standard deviation (SD). Categorical variables were expressed as frequencies and percentages. We employed one-way ANOVA for comparing continuous variables between groups, and Pearson’s Chi-square test for categorical variables. Statistical significance was set at a two-sided p-value < 0.05 for all analyses.

## Results

### Characteristics of the study population

**Fig. 1:**
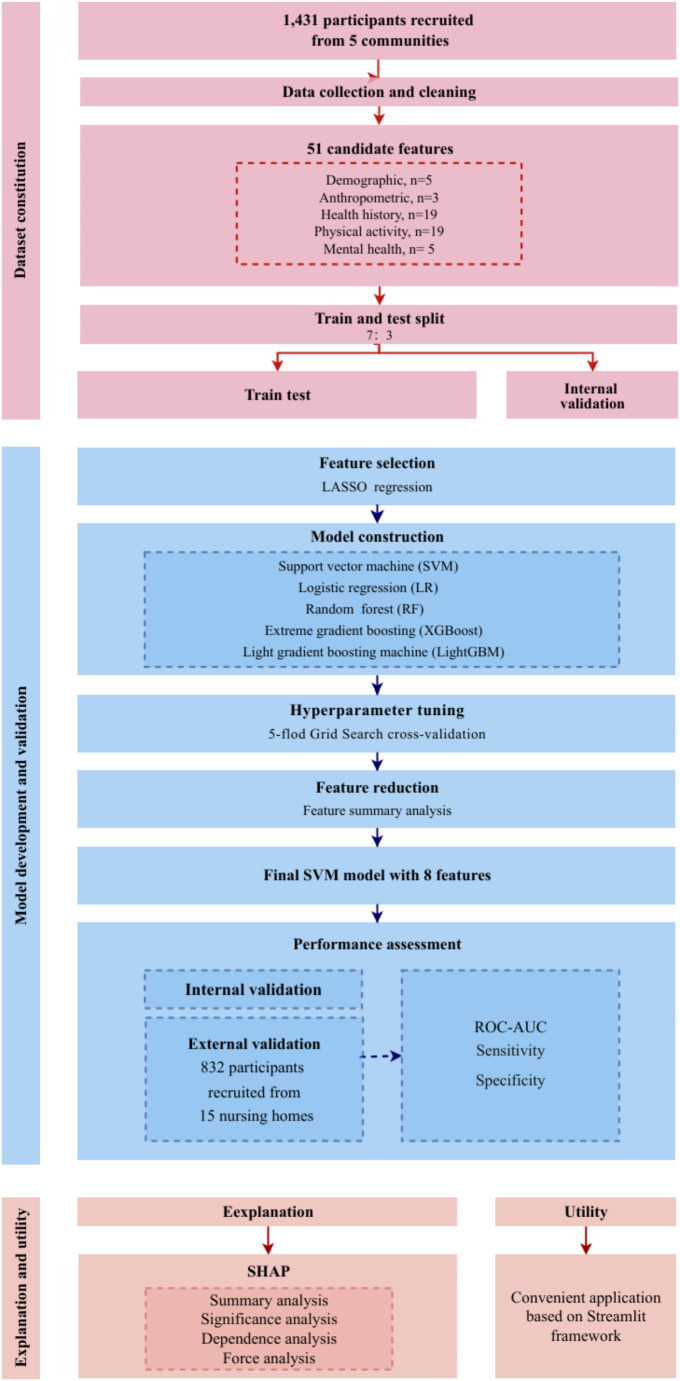
The flow chart of the study. SHAP, SHapley Additive exPlanations; LASSO, Least Absolute Shrinkage and Selection Operator

Supplementary Table 2 summarizes the characteristics of the study population. The training set from the Ziyang community cohort included 1,001 participants, of whom 121 (12.1%) were diagnosed with SO. The internal validation set included 430 participants, with 65 (15.1%) diagnosed with SO. The external validation set from Zigong nursing homes cohort included 832 residents, of whom 362 (43.5%) had SO. Figure 1 illustrates the study design in detail.

In the training and internal validation sets, significant differences were observed between SO and non-SO groups in several indicators, including age, BMI, neck circumference, waist circumference, thigh circumference, handgrip strength, and gait speed (*p* < 0.05). Similar differences were observed in the external validation set, except for calf circumference, polypharmacy, and chronic diseases. Notably, in the external validation set, the SO group had a significantly lower marriage rate than the non-SO group (14.9% vs. 34.3%, *p* < 0.001) and a higher proportion of low education level (85.4% vs. 68.1%, *p* < 0.001).

### Model development and performance comparison

#### Training set

We built five machine learning models based on the training set. Among these, XGBoost model performed best, followed by LightGBM, RF, LR, and SVM models (Figure S1 and Supplementary Table 3).

#### Internal validation set

**Fig. 2:**
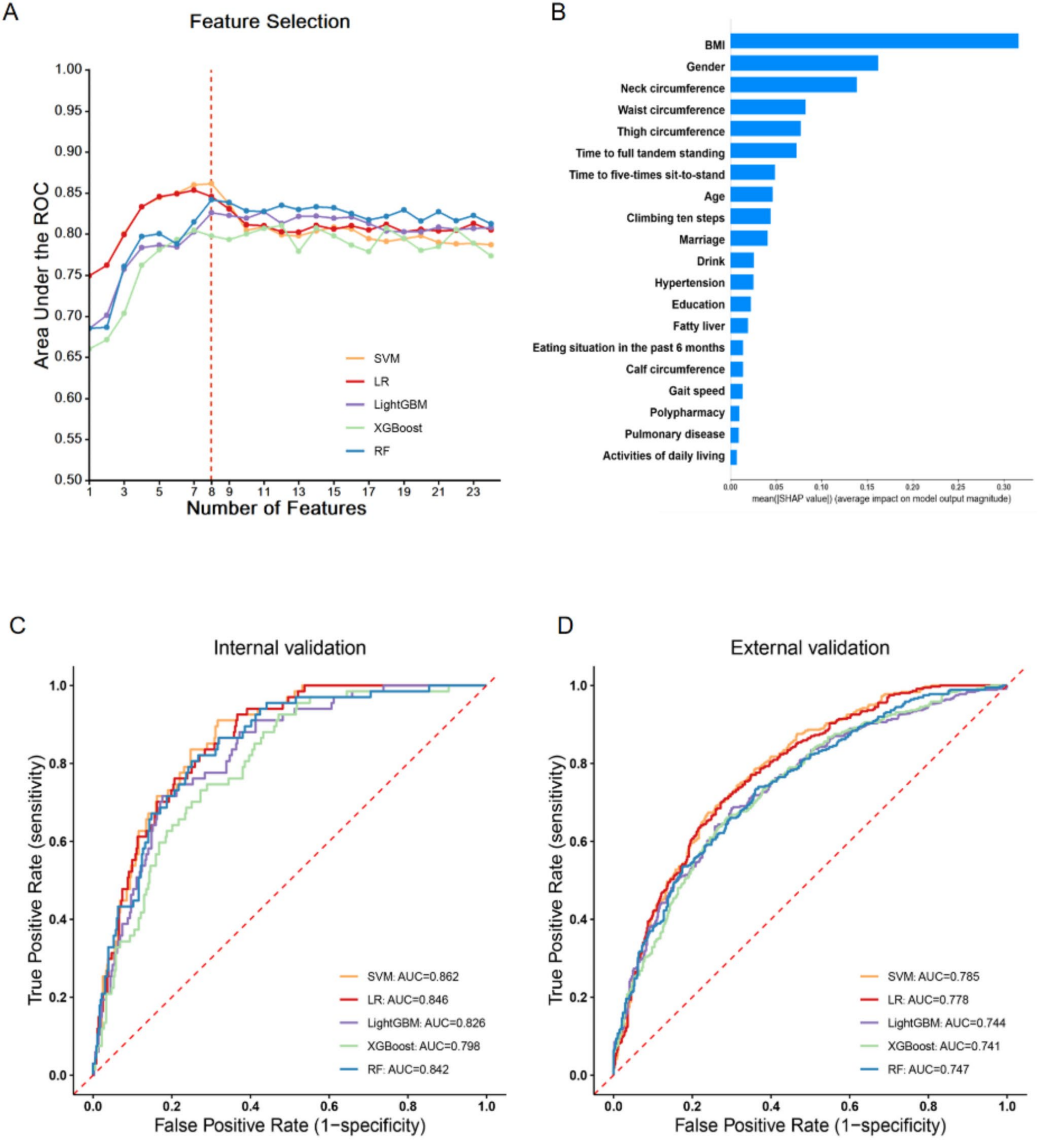
Performance of machine learning models to predict SO. (**A**) AUCs of the five machine learning models with different numbers of features. (**B**) The effect of each individual feature on model output and feature importance rank according to SHAP values. (**C**) AUCs of the five machine learning models based on 8-features in the internal validation set. (**D**) AUCs of the five machine learning models based on 8-features in the external validation set. AUC, area under the receiver operating characteristic curve; BMI, body mass index; LightGBM, light gradient boosting machine; LR, logistic regression; RF, random forest; SHAP, SHapley Additive exPlanations; SO, sarcopenic obesity; SVM, support vector machine; XGBoost, extreme gradient boosting

Figure 2A illustrates the changes in the AUCs for the five machine learning models with different numbers of features in the internal validation set. During the process of feature reduction based on the feature importance ranking, the 8-feature model demonstrated good predictive ability compared to models with other feature numbers. These 8 features include: BMI, gender, neck circumference, waist circumference, thigh circumference, time to full tandem standing (FTS), time to five-times sit-to-stand (FTSS), and age. Figure 2B presents the effect of each individual feature on model output and feature importance ranking.

When comparing the AUCs of these models based on eight features, the SVM model exhibited the highest performance (AUC = 0.862), followed by the LR model (AUC 0.846), the RF model (AUC = 0.842), the LightGBM model (AUC = 0.826), and the XGBoost model (AUC = 0.798) (Fig. 2C, Supplementary Tables 3, and Figure S2).

#### External validation set

Similar results were observed in the external validation set, with the SVM model maintaining the best performance (AUC = 0.785) (Fig. 2D, Supplementary Tables 3, and Figure S2). Given its superior performance in both internal and external validation sets, the SVM model was selected for further model explanation.

### Model explanation

**Fig. 3:**
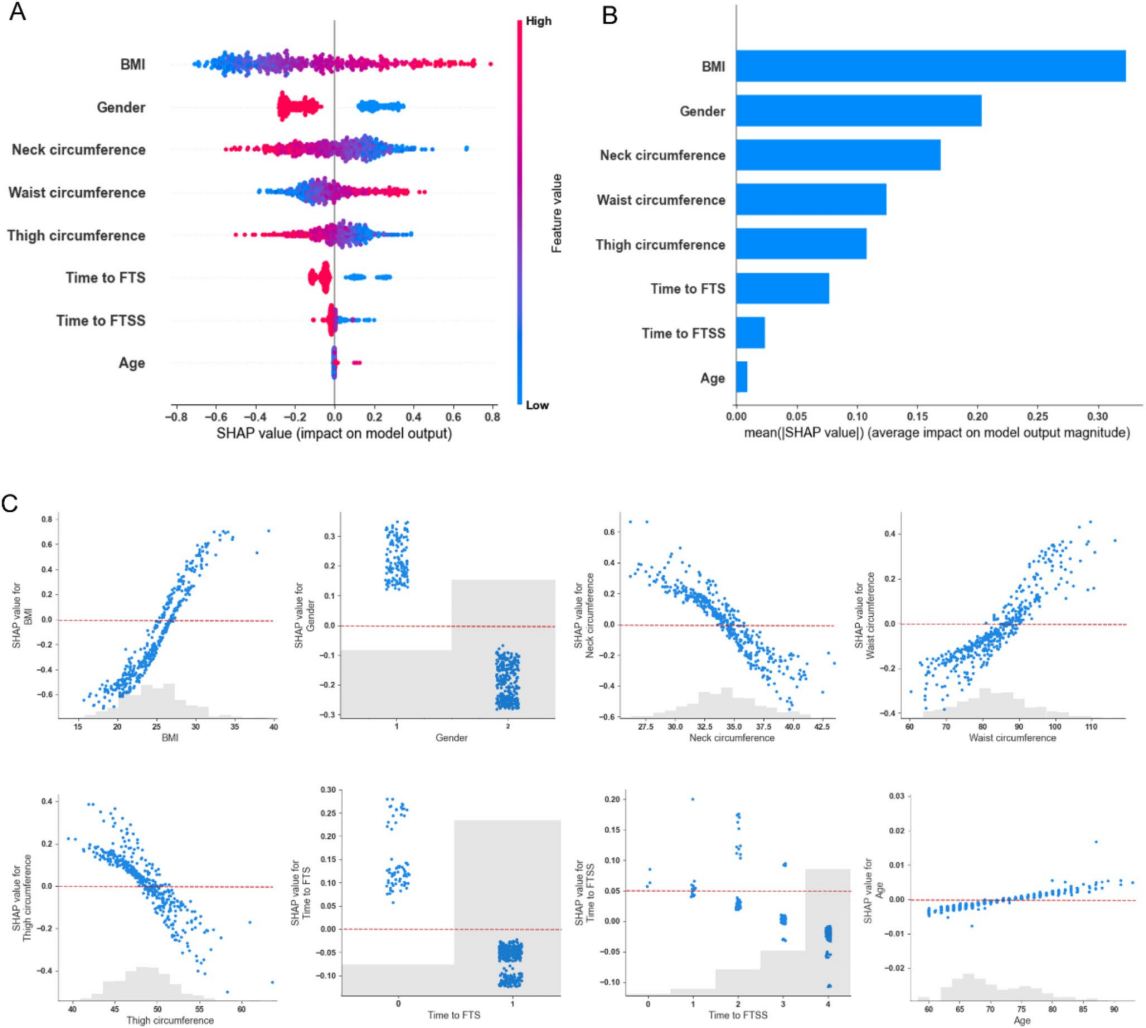
Global model explanation by the SHAP method in the internal validation set. (**A**) SHAP summary dot plot. (**B**) SHAP summary bar plot. Each dot represented a SHAP value for a feature of each individual. The colors of the dots indicate the values of the feature, with red representing higher feature values and blue representing lower feature values. (**C**) SHAP scatter plots. Each scatter plot showed the impact of each feature on model prediction. BMI, body mass index; FTS, time to full tandem standing; FTSS, time to five-times sit-to-stand; SHAP, SHapley Additive exPlanations

The SHAP approach was employed to interpret the output of the SVM model. Figure 3 presents the global model explanation in the internal validation set. The SHAP summary plots (Fig. 3A and B) revealed that BMI was the most important feature for SO prediction, followed by gender, neck circumference, waist circumference, thigh circumference, FTS, FTSS, and age.

The SHAP scatter plots (Fig. 3C) illustrate the impact of each feature on model prediction. Higher BMI, larger waist circumference, older age, male gender, longer time to FTS, and longer time to FTSS were associated with higher SHAP values, indicating a greater likelihood of SO. Conversely, larger neck circumference and thigh circumference were associated with lower SHAP values, suggesting a reduced likelihood of SO.

In the external validation set (Figure S3), the model explanation showed slight differences in feature importance ranking. Thigh circumference demonstrated greater importance than waist circumference, while the order of other features remained consistent (Figure S3A and S3B). The impact of each feature on model prediction was consistent with the internal validation set (Figure S3C).

### Explanation of machine learning model at individual level

**Fig. 4:**
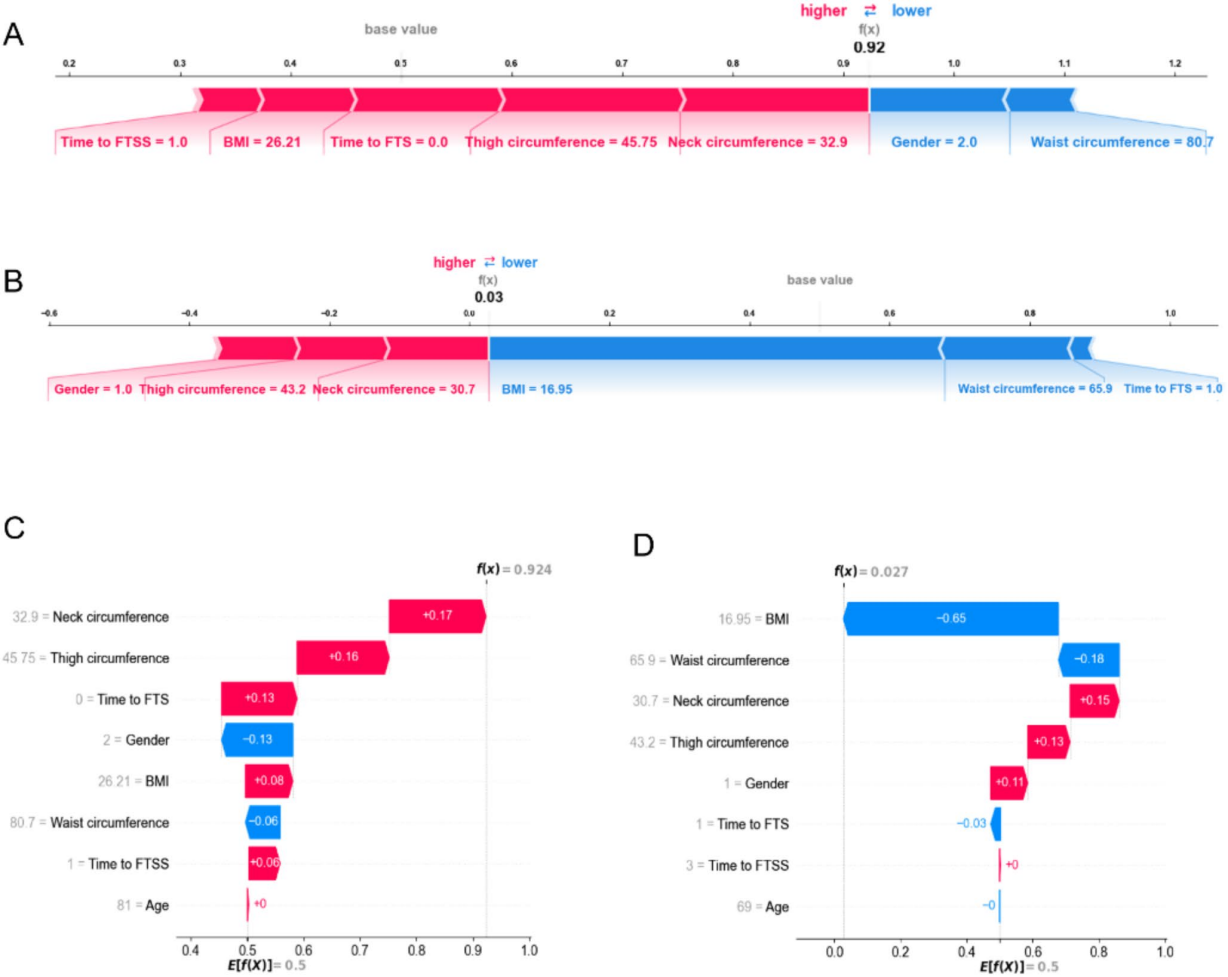
Local model explanation by the SHAP method in the internal validation set. (**A**) SHAP force plot of patient (A) (**B**) SHAP force plot of patient (B) The colors represented the contribution of each feature, with red increasing the possibility of SO and blue decreasing the possibility of SO. The longer of the color bar, the greater the contribution of the features. (**C**) SHAP waterfall plot of patient (A) (**D**) SHAP waterfall plot of patient (B) The SHAP waterfall plots visualized how each feature pushed the patient towards SO. BMI, body mass index; FTS, time to full tandem standing; FTSS, time to five-times sit-to-stand; SHAP, SHapley Additive exPlanations; SO, sarcopenic obesity

To demonstrate individual patient predictions, two cases from the internal validation set were randomly selected for local model explanation (Fig. 4). Patient A, an 81-year-old woman diagnosed with SO, had a predicted 92% risk of SO based on the SVM model. Age, time to FTSS, BMI, time to FTS, thigh circumference, and neck circumference increased the prediction of SO, while female gender and waist circumference decreased the prediction (Fig. 4A and C). Patient B, a 69-year-old man without SO, had a predicted 3% risk of SO. Lower BMI, smaller waist circumference, and shorter time to FTS contributed to reducing the risk of SO in this case (Fig. 4B and D).

Similar results of another two patients from the external validation set are shown in Figure S4.

### Clinical application

**Fig. 5:**
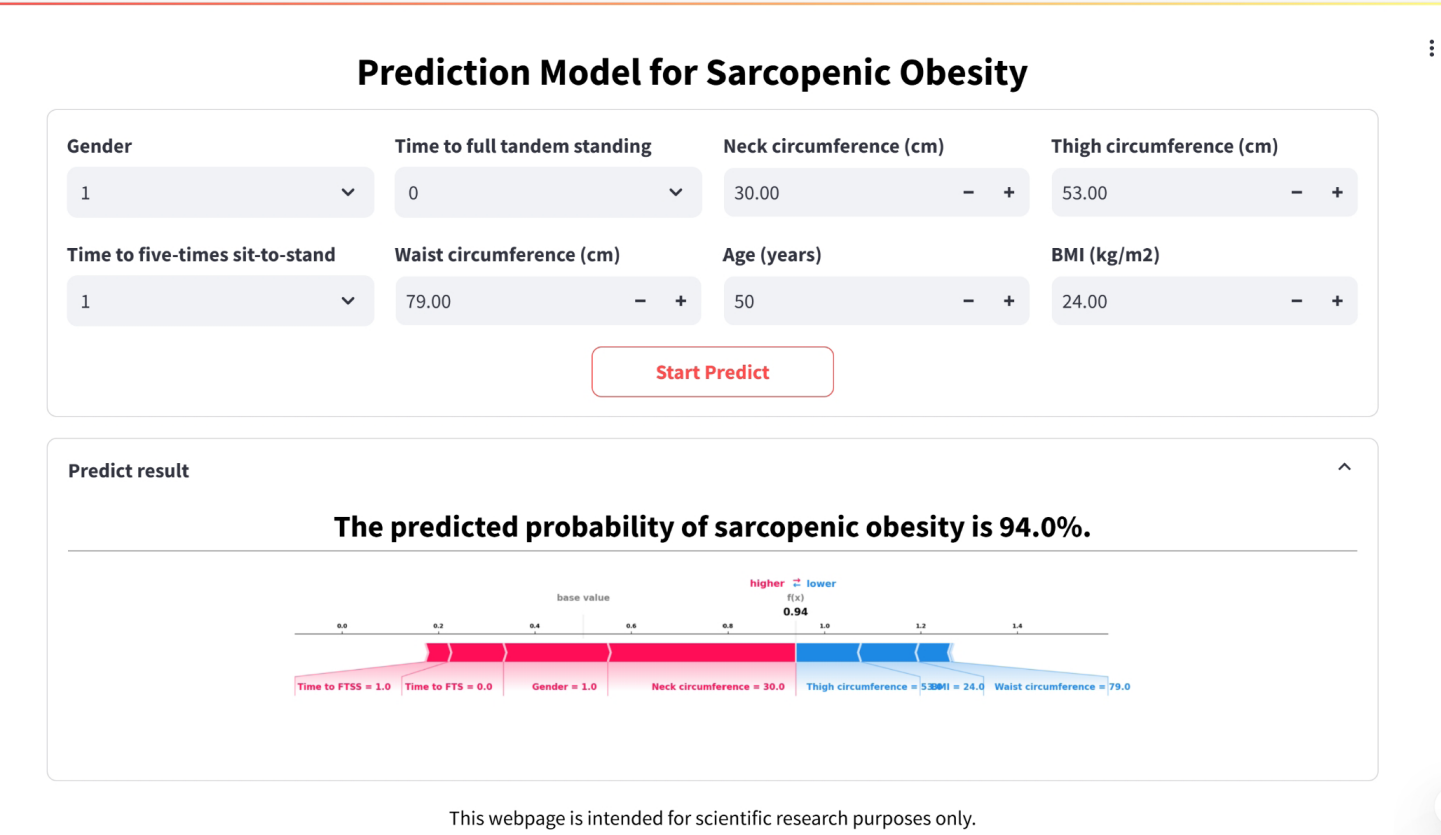
An example output of the web application. Gender: 1=men, 2=women; time to full tandem standing: 0 ≥10 s; 1 <10 s; time to five-times sit-to-stand: 0 >60 s or can’t finish, 1=16.70-60 s, 2=13.70-16.69 s, 3=11.20-13.69 s, 4 <11.20 s

To facilitate the clinical application of the SVM model, we developed a user-friendly web application (https://svcpredictapp.streamlit.app/). This application allows clinicians to input the values of the 8 features and automatically predicts the risk of SO for individual patients (Fig. 5). This tool can help healthcare professionals quickly identify potential SO patients for early intervention.

## Discussion

In this study, we developed and validated a machine learning-based model for predicting sarcopenic obesity (SO) in older adults. Our findings not only contribute to the understanding of SO but also offer a potentially valuable screening tool for clinical practice.

Among these five models tested (SVM, LR, RF, LightGBM, and XGBoost), the SVM model demonstrated superior performance in both the internal (AUC = 0.862) and external (AUC = 0.785) validation sets. The model identified eight key predictors of SO: BMI, gender, neck circumference, waist circumference, thigh circumference, FTS, FTSS, and age. These easily obtainable and economically accessible clinical characteristics could serve as valuable screening tools for SO in community and healthcare settings.

BMI emerged as the most crucial predictor in the SVM model. As widely recognized, BMI is a commonly used indicator of obesity. Evidence has indicated that obesity may precipitate the loss of muscle mass and function due to the negative impact of fat accumulation [3], eventually leading to SO. Moreover, individuals with obesity had a high prevalence of metabolism diseases, such as dyslipidemia [27], diabetes [28], cardiovascular disease [29], and metabolic associated fatty liver disease [30], which negatively impacted both anabolism and catabolism of muscle. Hence, our finding that a high BMI level was associated with SO aligns with these established conclusions.

In addition, other anthropometric parameters, including neck circumference, waist circumference, thigh circumference, were identified as relevant features of SO. Interestingly, we found that patients with elevated waist circumference was more likely to be SO, but higher neck circumference and thigh circumference showed an inverse relationship, suggesting that abdominal obesity may be a stronger predictor of SO. Similarly, studies by Luo et al. proved that individuals with SO showed shorter neck circumference [31]. However, Xu et al. [32] found that participants with elevated neck circumference had a higher proportion of pre-SO, regardless of adiposity status assessed by BMI, fat percentage, or visceral fat area. Currently, there is a lack of research regarding the relationship between thigh circumference and SO. These findings highlight the complex relationship between regional adiposity and muscle health, warranting further investigation into the underlying physiological mechanisms.

Existing evidence has shown that the prevalence of SO exhibited gender-specific variation. Batsis et al. [33] reported that individuals diagnosed with SO from the National Health and Nutrition Examination Survey 1999–2004 were more likely to be women. On the contrary, other studies reported a higher prevalence of SO in elderly man than in women [34, 35]. In line with the latter findings, our study indicated that men, rather than women, had an increased probability of SO. These discrepancies may be attributed to differences in study populations and SO definitions. Additionally, this may be related to the different pattern of body composition changes with aging between men and women. Du et al. [34] found that elderly men had more muscle mass than women, but experienced faster deterioration, while elderly women had more fat mass. Moreover, reduced testosterone [36] and estrogen [37] with aging exert detrimental effects on muscle biology and bone metabolism.

Age was identified as a significant feature of SO, consistent with previous research. Kemmler et al. [38] found that the prevalence of SO in community-dwelling women aged 70 years and older increased with aging. Similarly, research by Batsis et al. [33] indicated that the prevalence of SO in those aged over 80 years (women: 48.0%, men: 27.5%) was significantly higher those aged between 60 ~ 80 years (women: 25.1 ~ 36.0%, men: 6.7 ~ 14.9%). Furthermore, with the aging process, skeletal muscle function deteriorates quickly. Therefore, muscle functional parameters play a crucial role in the diagnosis of SO. In this study, we found that patients with SO had a poor performance in FTS and FTSS. FTS is a portion of the short physical performance battery (SPPB), a well-established instrument for measuring physical performance. Previous studies reported that FTS was an indicator of falls [39], postoperative complications [40], and other adverse health events [41]. FTSS is widely recognized as an indicator of low limbs strength, particularly in older adults [42]. A systematic review and meta-analysis have proved that FTSS was a reliable tool for assessing lower limbs strength, balance control, and mobility in both healthy and pathologic adults [43].

Our approach offers several advantages over existing machine learning models for SO prediction. While previous studies, such as those by Bae et al. [44] and Zambon et al. [45] have shown promising results, they often employed single algorithm designs without external validation and appeared to be resource-intensive. In contrast, our study compared multiple machine learning models and validated the results in two distinct populations (community-dwelling older adults and nursing home residents). This approach enhances the generalizability and cost-effectiveness of our model. Another strength of our study was the utilization of SHAP approach to overcome the “black box” nature of machine learning models, providing interpretability that is crucial for clinical applications.

To facilitate clinical use, we developed an easy-to-use web application based on the Streamlit framework, which enhances the accessibility of SO diagnosis for clinicians. By incorporating simple and readily available clinical features, this tool enables quick, real-time predictions without the need for specialized hardware or extensive computational resources. Its user-friendly design ensures seamless integration into routine clinical workflows, supporting early detection and intervention strategies for SO in various healthcare settings.

Our study has some limitations. Firstly, this model was exclusively drawn from Chinese populations, warranting caution in generalizing our results to global populations. However, the multi-center design, including both community and nursing home populations, lends to credibility to the generalizability of our results. Further studies based on more diverse populations are required to validate our model. Secondly, while we compared several machine learning models, future research could explore a wider range of algorithms. Thirdly, although our sample size provided reliable results, larger studies could further validate and refine our findings. Lastly, although our study, as a diagnostic test accuracy study, is appropriately designed as a cross-sectional study, future research with a longitudinal design is needed to further validate the model’s predictive ability and clinical utility.

## Conclusions

This study presents a novel, accessible, and interpretable approach to SO prediction using machine learning techniques. We successfully developed an SVM model with eight key predictors, demonstrating superior performance in both internal and external validation sets. The developed model and accompanying web application have the potential to enhance early detection and intervention strategies for SO in clinical practice.

Future research should focus on validating this model in diverse global populations, investigating the role of socioeconomic factors in SO prediction, and evaluating the impact of this tool on patient outcomes when integrated into comprehensive geriatric assessments. By enabling early identification of at-risk individuals, this approach may contribute to more timely interventions and improved health outcomes for older adults.

## Electronic supplementary material

Below is the link to the electronic supplementary material.

Supplementary Material 1

Supplementary Material 2

## Data Availability

No datasets were generated or analysed during the current study.

## Declarations

### Generative AI and AI-assisted technologies in the writing process

No Generative AI or AI-assisted technologies were used in the writing process.
