# Supplementary material for: Development and multi-center cross-setting validation of an explainable prediction model for sarcopenic obesity: a machine learning approach based on readily available clinical features
Source: Aging Clin Exp Res. 2025 Mar 1;37(1):63. doi: 10.1007/s40520-025-02975-z (PMC11870957; doi:10.1007/s40520-025-02975-z)
Supplement: Supplementary file 1 — Supplementary Material 1 [file 40520_2025_2975_MOESM1_ESM.docx]

Supplementary Table 1 The details of all variables and the measurement methods

| **Variables** | **Measurement methods** | |
| --- | --- | --- |
| **Demographic parameters** |  | |
| Age | By asking the question, 'How old are you?'. | |
| Gender |  | |
|  | 1 | Men |
|  | 2 | Women |
| Marital status |  | |
|  | 1 | Spinsterhood |
|  | 2 | Married |
|  | 3 | Divorced |
|  | 4 | Widowed |
| Education |  | |
|  | 1 | Primary school or below |
|  | 2 | Middle or high school |
|  | 3 | College or above |
| Smoking status |  | |
|  | 1 | Never smoked |
|  | 2 | Ever smoked |
|  | 3 | Current smoker |
| Drinking status |  | |
|  | 1 | Never drank |
|  | 2 | Ever drank |
|  | 3 | Current drinker |
| Polypharmacy 1 | Polypharmacy was defined as the concomitant use of five or more medications. | |
| Self-evaluation of nutrition |  | |
|  | 0 | Good |
|  | 1 | Bad or uncertain |
|  |  | |
| **Anthropometric parameters** |  | |
| Height | Body height was measured using a portable stadiometer to the nearest of 0.5 cm. | |
| Weight | Body weight was measured using a digital scale to the nearest of 0.1 kg. | |
| Body mass index (BMI) | BMI was calculated as the ratio of weight to the square of height (kg/m²). | |
| Neck circumference 2 | Neck circumference was measured below the laryngeal prominence in the neck, perpendicular to the largest neck axis. | |
| Mild-upper arm circumference 3 | Mild-upper arm circumference was measured at the midpoint of the tip of the shoulder (acromion process) and tip of the elbow (olecranon process). | |
| Chest circumference 4 | chest circumference was measured under the lower corners of the shoulder blades, and in front, at the level of attachment of four ribs to the sternum. | |
| Waist circumference 5 | Waist circumference was measured at the midpoint between the last palpable rib and the iliac crest. | |
| Hip circumference 5 | Hip circumference was measured at the widest part of the buttocks. | |
| Waist-hip ratio | The waist-hip ratio was calculated as the ratio of WC to HC. | |
| Thigh circumference 6 | Thigh circumference was measured directly below the gluteal fold. | |
| Calf circumference 6 | Calf circumference was measured at the thickest part of the calf. | |
|  |  | |
| **Chronic diseases** | By asking the question, 'Have you ever been diagnosed with the following diseases by doctors?'. | |
| Hypertension |  | |
| Diabetes |  |  |
| Pulmonary diseases |  |  |
| Coronary heart diseases |  |  |
| Stroke |  |  |
| Cancer |  |  |
| Fatty liver |  |  |
| Sleep disorders |  |  |
|  |  | |
| **Physical performance** |  | |
| SARC-F 7 |  | |
| Strength | By asking the question, 'how much difficulty do you have in lifting or carrying ten pounds?'. | |
|  | 0 | Not at all |
|  | 1 | Some |
|  | 2 | A lot or unable to finish |
| Assistance walking | By asking the question, 'how much difficulty do you have in walking across a room?'. | |
|  | 0 | Not at all |
|  | 1 | Some |
|  | 2 | A lot or unable to finish |
| Rising from a chair or a bed | By asking the question, 'How much difficulty do you have in transferring from a chair or a bed?'. | |
|  | 0 | Not at all |
|  | 1 | Some |
|  | 2 | A lot or unable to finish |
| Climbing stairs | By asking the question, 'How much difficulty do you have in climbing a flight of 10 stairs?'. | |
|  | 0 | Not at all |
|  | 1 | Some |
|  | 2 | A lot or unable to finish |
| Falls | By asking the question, 'How many times have you fallen in the past year?'. | |
|  | 0 | Not at all |
|  | 1 | Some |
|  | 2 | A lot or unable to finish |
| SPPB 8 |  | |
| Gait speed | A timed 4-m walk test at usual walking speed from a standing start was performed. | |
|  | 1 | >8.70 s |
|  | 2 | 6.21-8.70 s |
|  | 3 | 4.82-6.20 s |
|  | 4 | <4.82 s |
| Side-by-side standing | Participants were asked to remain standing with their feet as close together as possible, and time was recorded. | |
|  | 0 | <10 s |
|  | 1 | ≥10 |
| Semi tandem standing | Participants were asked to remain standing in a semi-tandem position, and time was recorded. | |
|  | 0 | <10 s |
|  | 1 | ≥10 |
| Full tandem standing (FTS) | Participants were asked to remain standing in a tandem position, and time was recorded. | |
|  | 0 | <10 s |
|  | 1 | ≥10 |
| Five-time sit-to-stand (FTSS) | Participants were asked to stand up and sit down five consecutive times as fast as safely possible, and time was recorded. | |
|  | 0 | >60 s or unable to finish |
|  | 1 | 16.70-60 s |
|  | 2 | 13.70-16.69 s |
|  | 3 | 11.20-13.69 s |
|  | 4 | <11.20 s |
| Activities of daily living (ADL) 9 |  | |
|  | 0 | Complete independently |
|  | 1 | Need help |
|  | 2 | Absolute dependence |
| Pick up things in standing position |  | |
|  | 0 | Able to pick up things safely and easily |
|  | 1 | Need supervision |
|  | 2 | Unable to pick up things, but can reach the position 2-5cm away from the things |
|  | 3 | Unable to pick up things and needs monitoring when trying |
|  | 4 | Unable to attempt |
| Self-report of activities in the last 1 month |  | |
|  | 0 | Normal |
|  | 1 | Less active than usual |
|  | 2 | Stay in bed or wheelchair less than half a day |
|  | 3 | Stay in bed or wheelchair for most of the time/completely bedridden |
|  |  | |
| **Mental health** |  | |
| Life satisfaction | By asking the question, 'Are you satisfied with your life?'. | |
|  | 0 | Yes |
|  | 1 | No |
| Life empty | By asking the question, 'Are you often get bored?'. | |
|  | 0 | No |
|  | 1 | Yes |
| Feel happy | By asking the question, 'Do you often feel happy?'. | |
|  | 0 | Yes |
|  | 1 | No |
| Feel isolated | By asking the question, 'Do you often feel isolated?'. | |
|  | 0 | No |
|  | 1 | Yes |
| A meaningless life | By asking the question, 'Do you feel pretty worthless the way you are now?'. | |
|  | 0 | No |
|  | 1 | Yes |
| References:  1. Masnoon N, Shakib S, Kalisch-Ellett L, Caughey GE. What is polypharmacy? A systematic review of definitions. BMC Geriatr. 2017;17(1):230. 2. Filgueiras MS, Albuquerque FM, Castro APP, Rocha NP, Milagres LC, Novaes JF. Neck circumference cutoff points to identify excess android fat. J Pediatr (Rio J). 2020;96(3):356-63. 3. Zhu Y, Lin Q, Zhang Y, Deng H, Hu X, Yang X, et al. Mid-upper arm circumference as a simple tool for identifying central obesity and insulin resistance in type 2 diabetes. PLoS One. 2020;15(5): e0231308. 4. Safonicheva O, Zaborova V, Lazareva I, Kryuchkova K, Bolotskaya A, Ovchinnikova M, et al. Age-Related Study of Anthropometry Indicators, Body Composition, Strength and Vital Capacity at Masters Athletics: How to Postpone Sarcopenia. Clin Interv Aging. 2023; 18:2155-64. 5. Tang H, Li R, Li R, Lian R, Chen X, Jiang W, et al. Sarcopenic obesity in nursing home residents: a multi-center study on diagnostic methods and their association with instrumental activities of daily living. BMC Geriatr. 2024;24(1):446. 6. Yoon MK, Kang JG, Lee SJ, Ihm SH, Huh KB, Kim CS. Relationships between Thigh and Waist Circumference, Hemoglobin Glycation Index, and Carotid Plaque in Patients with Type 2 Diabetes. Endocrinol Metab (Seoul). 2020;35(2):319-28. 7. Nishikawa H, Asai A, Fukunishi S, Takeuchi T, Goto M, Ogura T, et al. Screening Tools for Sarcopenia. In Vivo. 2021;35(6):3001-9. 8. Lauretani F, Ticinesi A, Gionti L, Prati B, Nouvenne A, Tana C, et al. Short-Physical Performance Battery (SPPB) score is associated with falls in older outpatients. Aging Clin Exp Res. 2019;31(10):1435-42. 9. Nakanishi K, Yamaga T, Ikeya M. Gaps between Activities of Daily Living Performance and Capacity in People with Mild Dementia. Int J Environ Res Public Health. 2022;19(23). | | |

Supplementary Table 2. Characteristics of the study population

| Characteristic | Training set | | |  | | Internal validation set | | | | | |  | | External validation set | | | | | | |  |
| --- | --- | --- | --- | --- | --- | --- | --- | --- | --- | --- | --- | --- | --- | --- | --- | --- | --- | --- | --- | --- | --- |
|  | (N = 1, 001) | | |  | | (N = 430) | | | | | |  | | (N = 832) | | | | | | |  |
|  | SO | Non-SO | *P ^a^* |  | | SO | | Non-SO | | *P ^a^* | |  | | SO | | Non-SO | | *P ^a^* | | |  |
|  | (N = 121) | (N = 880) |  |  | | (N = 65) | | (N = 365) | |  |  |  | | (N = 362) | | (N = 470) | |  |  |  |  |
| Demographic information | | | | | | | | | | | | | | | | | | | | | |
| Women, n (%) | 66 (54.5) | 613 (69.7) | .001 |  | | 40 (61.5) | | 238 (65.2) | | .67 | |  | | 99 (27.3) | | 197 (41.9) | | <.001 | | |  |
| Age, years, Mean (SD) | 74.3 (7.5) | 70.5 (6.3) | <.001 |  | | 75.1 (6.7) | | 70.1 (6.2) | | <.001 | |  | | 76.8 (8.2) | | 73.5 (8.6) | | <.001 | | |  |
| Current smoker, n (%) | 11 (9.1) | 81 (9.2) | .99 |  | | 7 (10.8) | | 42 (11.5) | | .99 | |  | | 116 (32.0) | | 156 (33.2) | | .78 | | |  |
| Current drinker, n (%) | 35 (28.9) | 225 (25.6) | .50 |  | | 9 (13.8) | | 90 (24.7) | | .08 | |  | | 79 (21.8) | | 111 (23.6) | | .60 | | |  |
| Education (≤6 years), n (%) | 79 (65.3) | 519 (59.0) | .22 |  | | 43 (66.2) | | 202 (55.3) | | .14 | |  | | 309 (85.4) | | 320 (68.1) | | <.001 | | |  |
| Married, n (%) | 89 (73.6) | 662 (75.2) | .77 |  | | 40 (61.5) | | 282 (77.3) | | .01 | |  | | 54 (14.9) | | 161 (34.3) | | <.001 | | |  |
| Polypharmacy, n (%) | 15 (12.4) | 65 (7.4) | .08 |  | | 14 (21.5) | | 19 (5.2) | | <.001 | |  | | 42(11.6) | | 59 (12.6) | | .76 | | |  |
| Self-evaluation of nutrition | | | .99 |  | |  | |  | | .25 | |  | |  | |  | | .07 | | | |
| Good | 109 (90.1) | 795 (90.3) |  |  | | 54 (83.1) | | 325 (89.0) | |  | |  | | 246 (68.0) | | 348 (74.0) | |  | |  |  |
| Bad or uncertain | 12 (9.9) | 85 (9.7) |  |  | | 11 (16.9) | | 40 (11.0) | |  | |  | | 116 (32.0) | | 122 (26.0) | |  | |  |  |
| Anthropometric information | | | | | | | | | | | | | | | | | | | | | |
| Height, m, Mean (SD) | 1.58 (0.08) | 1.55 (0.08) | .96 | |  | | 1.56 (0.07) | | 1.56 (0.08) | | .94 | |  | | 1.52 (0.10) | | 1.56 (0.09) | | <.001 | | |
| Weight, kg, Mean (SD) | 66.5 (10.2) | 59.4 (10.6) | <.001 | |  | | 66.8 (10.2) | | 58.9 (10.5) | | <.001 | |  | | 57.8 (9.7) | | 55.9 (10.2) | | .007 | | |
| BMI, kg/m^2^, Mean (SD) | 27.5 (3.3) | 24.54 (3.6) | <.001 | |  | | 27.5 (3.1) | | 24.3 (3.6) | | <.001 | |  | | 25.1 (3.4) | | 23.0 (3.7) | | <.001 | | |
| Neck circumference, cm, Mean (SD) | 35.7 (2.9) | 34.1 (3.1) | <.001 | |  | | 35.4 (2.7) | | 34.0 (3.0) | | .001 | |  | | 34.0 (3.0) | | 33.3 (3.3) | | .007 | | |
| Mid-upper arm circumference, cm, Mean (SD) | 30.4 (2.9) | 30.0 (3.0) | .24 | |  | | 30.9 (3.0) | | 30.2 (3.2) | | .09 | |  | | 30.1 (2.8) | | 28.8 (3.3) | | <.001 | | |
| Chest circumference, cm, Mean (SD) | 90.9 (6.8) | 90.1 (6.7) | .21 | |  | | 92.2 (6.9) | | 90.5 (7.2) | | .08 | |  | | 89.1 (6.3) | | 87.8 (7.4) | | .007 | | |
| Waist circumference, cm, Mean (SD) | 89.2 (8.2) | 83.0 (9.4) | <.001 | |  | | 89.0 (7.9) | | 82.6 (9.6) | | <.001 | |  | | 88.2 (8.9) | | 84.5 (9.9) | | <.001 | | |
| Hip circumference, cm, Mean (SD) | 94.0 (5.2) | 93.1 (5.4) | .12 | |  | | 94.7 (5.5) | | 93.4 (6.1) | | .11 | |  | | 95.4 (7.2) | | 94.0 (7.7) | | .008 | | |
| Waist-hip ratio, Mean (SD) | 0.89 (0.05) | 0.89 (0.06) | .76 | |  | | 0.90 (0.07) | | 0.90 (0.06) | | .37 | |  | | 0.93 (0.07) | | 0.90 (0.07) | | <.001 | | |
| Thigh circumference, cm, Mean (SD) | 49.9 (2.9) | 48.6 (3.4) | <.001 | |  | | 50.2 (3.4) | | 48.2 (3.2) | | <.001 | |  | | 47.5 (3.1) | | 46.8 (3.4) | | .005 | | |
| Calf circumference, cm, Mean (SD) | 34.2 (2.6) | 33.3 (2.9) | .002 | |  | | 34.2 (2.9) | | 33.1 (2.7) | | .005 | |  | | 32.1 (2.8) | | 32.4 (3.3) | | .21 | | |
| Chronic diseases | | | | | | | | | | | | | | | | | | | | | |
| Hypertension, n (%) | 59 (48.8) | 298 (33.9) | .002 | |  | | 38 (58.5) | | 117 (32.1) | | <.001 | |  | | 141 (39.0) | | 175 (37.2) | | .67 | | |
| Diabetes, n (%) | 29 (24.0) | 138 (15.7) | .03 | |  | | 15 (23.1) | | 44 (12.1) | | .03 | |  | | 59 (16.3) | | 75 (16.0) | | .97 | | |
| Pulmonary diseases, n (%) | 17 (14.0) | 74 (8.4) | .06 | |  | | 9 (13.8) | | 31 (8.5) | | .26 | |  | | 50 (13.8) | | 58 (12.3) | | .60 | | |
| Coronary heart diseases, n (%) | 16 (13.2) | 57 (6.5) | .01 | |  | | 9 (13.8) | | 22 (6.0) | | .047 | |  | | 27 (7.5) | | 35 (7.4) | | .99 | | |
| Stroke, n (%) | 7 (5.8) | 39 (4.4) | .66 | |  | | 4 (6.2) | | 17 (4.7) | | .84 | |  | | 15 (4.1) | | 24 (5.1) | | .63 | | |
| Cancer, n (%) | 4 (3.3) | 11 (1.2) | .18 | |  | | 1 (1.5) | | 1 (0.3) | | .70 | |  | | 1 (0.3) | | 4 (0.9) | | .54 | | |
| Fatty liver, n (%) | 5 (4.1) | 71 (8.1) | .18 | |  | | 5 (7.7) | | 20 (5.5) | | .68 | |  | | 2 (0.6) | | 6 (1.3) | | .48 | | |
| Sleep disorders, n (%) | 9 (7.4) | 48 (5.5) | .50 | |  | | 7 (10.8) | | 26 (7.1) | | .45 | |  | | 27 (7.5) | | 50 (10.6) | | .15 | | |
| Physical activities | | | | | | | | | | | | | | | | | | | | | |
| SARC-F |  |  |  | |  | |  | |  | |  | |  | |  | |  | |  | | |
| Strength |  |  | .11 | |  | |  | |  | | .16 | |  | |  | |  | | .04 | | |
| 0 points | 99 (81.8) | 760 (86.4) |  | |  | | 50 (76.9) | | 314 (86.0) | |  | |  | | 237 (65.5) | | 340 (72.3) | |  | | |
| 1 point | 13 (10.7) | 89 (10.1) |  | |  | | 11 (16.9) | | 35 (9.6) | |  | |  | | 73 (20.2) | | 65 (13.8) | |  | | |
| 2 points | 9 (7.4) | 31 (3.5) |  | |  | | 4 (6.2) | | 16 (4.4) | |  | |  | | 52 (14.4) | | 65 (13.8) | |  | | |
| Assistance walking |  |  | .05 | |  | |  | |  | | .84 | |  | |  | |  | | .14 | | |
| 0 points | 113 (93.4) | 850 (96.6) |  | |  | | 62 (95.4) | | 346 (94.8) | |  | |  | | 283 (78.2) | | 393 (83.6) | |  | | |
| 1 point | 8 (6.6) | 24 (2.7) |  | |  | | 3 (4.6) | | 17 (4.7) | |  | |  | | 65 (18.0) | | 63 (13.4) | |  | | |
| 2 points | 0 (0.0) | 6 (0.7) |  | |  | | 0 (0.0) | | 2 (0.5) | |  | |  | | 14 (3.9) | | 14 (3.0) | |  | | |
| Rising from a chair or a bed | | | .02 | |  | |  | |  | | .22 | |  | |  | |  | | .08 | | |
| 0 points | 105 (86.8) | 820 (93.2) |  | |  | | 59 (90.8) | | 347 (95.1) | |  | |  | | 280 (77.3) | | 391 (83.2) | |  | | |
| 1 point | 16 (13.2) | 55 (6.2) |  | |  | | 6 (9.2) | | 16 (4.4) | |  | |  | | 72 (19.9) | | 72 (15.3) | |  | | |
| 2 points | 0 (0.0) | 5 (0.6) |  | |  | | 0 (0.0) | | 2 (0.5) | |  | |  | | 10 (2.8) | | 7 (1.5) | |  | | |
| Climbing stairs |  |  | .11 | |  | |  | |  | | .14 | |  | |  | |  | | <.001 | | |
| 0 points | 97 (80.2) | 749 (85.1) |  | |  | | 52 (80.0) | | 314 (86.0) | |  | |  | | 211 (58.3) | | 342 (72.8) | |  | | |
| 1 point | 18 (14.9) | 113 (12.8) |  | |  | | 12 (18.5) | | 38 (10.4) | |  | |  | | 107 (29.6) | | 88 (18.7) | |  | | |
| 2 points | 6 (5.0) | 18 (2.0) |  | |  | | 1 (1.5) | | 13 (3.6) | |  | |  | | 44 (12.2) | | 40 (8.5) | |  | | |
| Falls, n (%) | 28 (23.1) | 145 (16.5) | .09 | |  | | 16 (24.6) | | 46 (12.6) | | .02 | |  | | 66 (18.2) | | 72 (15.3) | | .31 | | |
| SPPB |  |  |  | |  | |  | |  | |  | |  | |  | |  | |  | | |
| Gait speed, m/s, Mean (SD) | 1.4 (0.5) | 1.2 (0.4) | <.001 | |  | | 1.5 (0.6) | | 1.2 (0.4) | | <.001 | |  | | 0.8 (0.3) | | 0.9 (0.3) | | <.001 | | |
| Side-by-side standing | | | .04 | |  | |  | |  | | 0.50 | |  | |  | |  | | .66 | | |
| 0 points | 117 (96.7) | 873 (99.2) |  | |  | | 63 (96.9) | | 361 (98.9) | |  | |  | | 350 (96.7) | | 458 (97.4) | |  | | |
| 1 point | 4 (3.3) | 7 (0.8) |  | |  | | 2 (3.1) | | 4 (1.1) | |  | |  | | 12 (3.3) | | 12 (2.6) | |  | | |
| Semi tandem standing | | | .03 | |  | |  | |  | | .03 | |  | |  | |  | | .12 | | |
| 0 points | 115 (95.0) | 866 (98.4) |  | |  | | 59 (90.8) | | 355 (97.3) | |  | |  | | 321 (88.7) | | 433 (92.1) | |  | | |
| 1 point | 6 (5.0) | 14 (1.6) |  | |  | | 6 (9.2) | | 10 (2.7) | |  | |  | | 41 (11.3) | | 37 (7.9) | |  | | |
| Full tandem standing | | | <.001 | |  | |  | |  | | .001 | |  | |  | |  | | <.001 | | |
| 0 points | 37 (30.6) | 91 (10.3) |  | |  | | 20 (30.8) | | 51 (14.0) | |  | |  | | 131 (36.2) | | 94(20.0) | |  | | |
| 1 point | 84 (69.4) | 789 (89.7) |  | |  | | 45 (69.2) | | 314 (86.0) | |  | |  | | 231 (63.8) | | 376 (80.0) | |  | | |
| Five-time sit-to-stand | | | .06 | |  | |  | |  | | <.001 | |  | |  | |  | | <.001 | | |
| 0 points | 1 (0.8) | 4 (0.5) |  | |  | | 1 (0.2) | | 2 (0.5) | |  | |  | | 110 (30.4) | | 207 (44.0) | |  | | |
| 1 point | 9 (7.4) | 30 (3.4) |  | |  | | 8 (12.3) | | 7 (1.9) | |  | |  | | 72 (19.9) | | 104 (22.1) | |  | | |
| 2 points | 15 (12.4) | 101 (11.5) |  | |  | | 15 (23.1) | | 41 (11.2) | |  | |  | | 75 (20.7) | | 68 (14.5) | |  | | |
| 3 points | 32 (26.4) | 178 (20.2) |  | |  | | 19 (29.2) | | 85 (23.3) | |  | |  | | 77 (21.3) | | 71 (15.1) | |  | | |
| 4 points | 64 (52.9) | 567 (64.4) |  | |  | | 22 (33.8) | | 230 (63.0) | |  | |  | | 28 (7.7) | | 20 (4.3) | |  | | |
| Activities of daily living | | | .38 | |  | |  | |  | | .79 | |  | |  | |  | | .10 | | |
| 0 points | 119 (98.3) | 869 (98.8) |  | |  | | 64 (98.5) | | 355 (97.3) | |  | |  | | 297 (82.0) | | 407 (86.6) | |  | | |
| 1 point | 2 (1.7) | 6 (0.7) |  | |  | | 1 (1.5) | | 8 (2.2) | |  | |  | | 57 (15.7) | | 59 (12.6) | |  | | |
| 2 points | 0 (0.0) | 5 (0.6) |  | |  | | 0 (0.0) | | 2 (0.5) | |  | |  | | 8 (2.2) | | 4 (0.9) | |  | | |
| Pick up things in standing position | | | .01 | |  | |  | |  | | .50 | |  | |  | |  | | .004 | | |
| 0 points | 105 (86.8) | 835 (94.9) |  | |  | | 60 (92.3) | | 346 (94.8) | |  | |  | | 256 (70.7) | | 377 (80.2) | |  | | |
| 1 point | 14 (11.6) | 37 (4.2) |  | |  | | 5 (7.7) | | 13 (3.6) | |  | |  | | 74 (20.4) | | 67 (14.3) | |  | | |
| 2 points | 0 (0.0) | 1 (0.1) |  | |  | | 0 (0.0) | | 1 (0.3) | |  | |  | | 16 (4.4) | | 6 (1.3) | |  | | |
| 3 points | 1 (0.8) | 4 (0.5) |  | |  | | 0 (0.0) | | 4 (1.1) | |  | |  | | 7 (1.9) | | 11 (2.3) | |  | | |
| 4 points | 1 (0.8) | 3 (0.3) |  | |  | | 0 (0.0) | | 1 (0.3) | |  | |  | | 9 (2.5) | | 9 (1.9) | |  | | |
| Self-report of activities in the last 1 month | | | .73 | |  | |  | |  | | .01 | |  | |  | |  | | .15 | | |
| 0 points | 118 (97.5) | 865 (98.3) |  | |  | | 60 (92.3) | | 359 (98.4) | |  | |  | | 330 (91.2) | | 447 (95.1) | |  | | |
| 1 point | 3 (2.5) | 12 (1.4) |  | |  | | 3 (4.6) | | 4 (1.1) | |  | |  | | 27 (7.5) | | 20 (4.3) | |  | | |
| 2 points | 0 (0.0) | 1 (0.1) |  | |  | | 0 (0.0) | | 1 (0.3) | |  | |  | | 1 (0.3) | | 1 (0.2) | |  | | |
| 3 points | 0 (0.0) | 2 (0.2) |  | |  | | 2 (3.1) | | 1 (0.3) | |  | |  | | 4 (1.1) | | 2 (0.4) | |  | | |
| Mental health | | | | | | | | | | | | | | | | | | | | | |
| Life satisfaction, n (%) | 119 (98.3) | 872 (99.1) | .78 | |  | | 65 (100.0) | | 364 (99.7) | | .99 | |  | | 356 (98.3) | | 465 (98.9) | | .66 | | |
| Life empty, n (%) | 9 (7.4) | 41 (4.7) | .27 | |  | | 3 (4.6) | | 18 (4.9) | | .99 | |  | | 21 (5.8) | | 30 (6.4) | | .84 | | |
| Feel happy, n (%) | 117 (96.7) | 867 (98.5) | .28 | |  | | 65 (100.0) | | 360 (98.6) | | .75 | |  | | 348 (96.1) | | 459 (97.7) | | .28 | | |
| Feel isolated, n (%) | 11 (9.1) | 32 (3.6) | .01 | |  | | 4 (6.2) | | 13 (3.6) | | .52 | |  | | 27 (7.5) | | 21 (4.5) | | .09 | | |
| A meaningless life, n (%) | 7 (5.8) | 51 (5.8) | .99 | |  | | 2 (3.1) | | 14 (3.8) | | .99 | |  | | 30 (8.3) | | 30 (6.4) | | .36 | | |

BMI, body mass index; SD, standard deviation; SO, sarcopenic obesity. The bold values indicate p<0.05.

* One-way ANOVA test; Pearson's Chi-squared tes

Supplementary Table 3. Evaluation of model performance

|  | **AUC** | **Sensitivity** | **Specificity** | **PPV** | **NPV** | **Accuracy** | **F1-score** | **Calibration** |
| --- | --- | --- | --- | --- | --- | --- | --- | --- |
|  |  | **(Recall)** |  | **(Precision)** |  |  |  |  |
| **Training set** | | | | | |  |  |  |
| SVM | 0.901 | 0.825 | 0.833 | 0.832 | 0.827 | 0.828 | 0.827 | 0.126 |
| LR | 0.902 | 0.822 | 0.838 | 0.835 | 0.825 | 0.829 | 0.828 | 0.125 |
| LightGBM | 0.998 | 0.99 | 0.968 | 0.969 | 0.99 | 0.979 | 0.979 | 0.021 |
| XGBoost | 0.999 | 0.999 | 0.998 | 0.998 | 0.999 | 0.997 | 0.997 | 0.006 |
| RF | 0.989 | 0.955 | 0.939 | 0.94 | 0.954 | 0.945 | 0.946 | 0.058 |
| **Internal validation set** | | | | | |  |  |  |
| SVM | 0.862 | 0.731 | 0.807 | 0.432 | 0.942 | 0.81 | 0.543 | 0.13 |
| LR | 0.846 | 0.701 | 0.832 | 0.435 | 0.938 | 0.812 | 0.537 | 0.138 |
| LightGBM | 0.826 | 0.493 | 0.89 | 0.452 | 0.905 | 0.828 | 0.471 | 0.172 |
| XGBoost | 0.798 | 0.373 | 0.884 | 0.373 | 0.884 | 0.805 | 0.373 | 0.139 |
| RF | 0.842 | 0.507 | 0.883 | 0.44 | 0.907 | 0.823 | 0.472 | 0.142 |
| **External validation set** | | | | | |  |  |  |
| SVM | 0.785 | 0.888 | 0.494 | 0.574 | 0.849 | 0.665 | 0.697 | 0.236 |
| LR | 0.778 | 0.876 | 0.49 | 0.56 | 0.83 | 0.647 | 0.683 | 0.242 |
| LightGBM | 0.744 | 0.47 | 0.855 | 0.714 | 0.678 | 0.688 | 0.567 | 0.313 |
| XGBoost | 0.741 | 0.448 | 0.849 | 0.695 | 0.666 | 0.674 | 0.545 | 0.256 |
| RF | 0.747 | 0.495 | 0.847 | 0.713 | 0.685 | 0.694 | 0.584 | 0.256 |

AUC, the area under the receiver operating characteristic curve; LightGBM , light gradient boosting machine; LR, logistic regression; NPV, negative predictive value; PPV, positive predictive value; RF, random forest; SVM, support vector machine; XGBoost, extreme gradient boosting.
