# Supplementary material for: Development and multi-center cross-setting validation of an explainable prediction model for sarcopenic obesity: a machine learning approach based on readily available clinical features
Source: Aging Clin Exp Res. 2025 Mar 1;37(1):63. doi: 10.1007/s40520-025-02975-z (PMC11870957; doi:10.1007/s40520-025-02975-z)

**Figure S1.** AUCs of the five machine learning models based on 8-features in the training set.


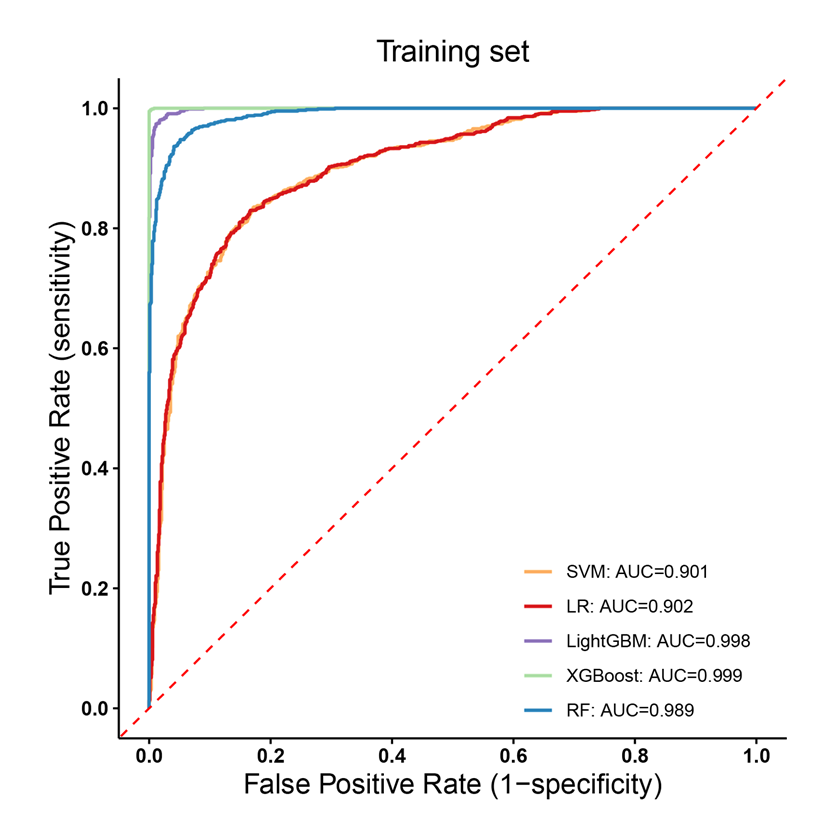


**Figure S2.** Calibration curves and decision curve analysis of the five machine learning models based on 8-features in the internal validation and external validation. (A) Decision curve analysis of the five machine learning models based on 8-features in the internal validation. (B) Decision curve analysis of the five machine learning models based on 8-features in the external validation. (C) Calibration curves of the five machine learning models based on 8-features in the internal validation. (D) Calibration curves of the five machine learning models based on 8-features in the external validation.

**
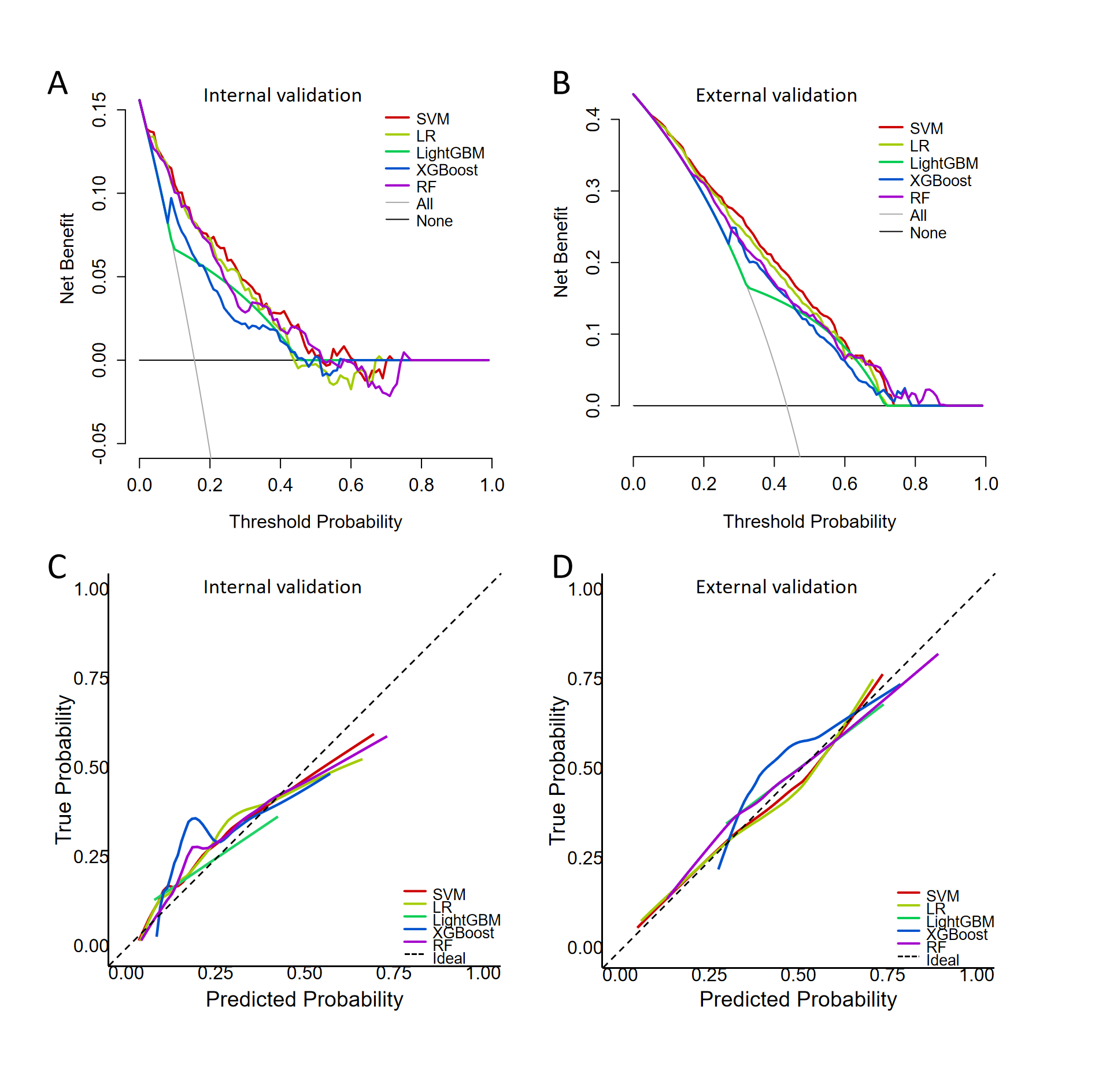
**

**Figure S3.** Global model explanation by the SHAP method in the external validation set. (A) SHAP summary dot plot. (B) SHAP summary bar plot. Each dot represents a SHAP value for a feature of each individual patient. The colors of the dots indicate the values of the feature, with red representing higher feature values and blue representing lower feature values. (C) SHAP scatter plots. Each scatter plot showed the impact of each feature on model prediction. BMI, body mass index; FTS, time to full tandem standing; FTSS, time to five-times sit-to-stand; SHAP, SHapley Additive exPlanations.


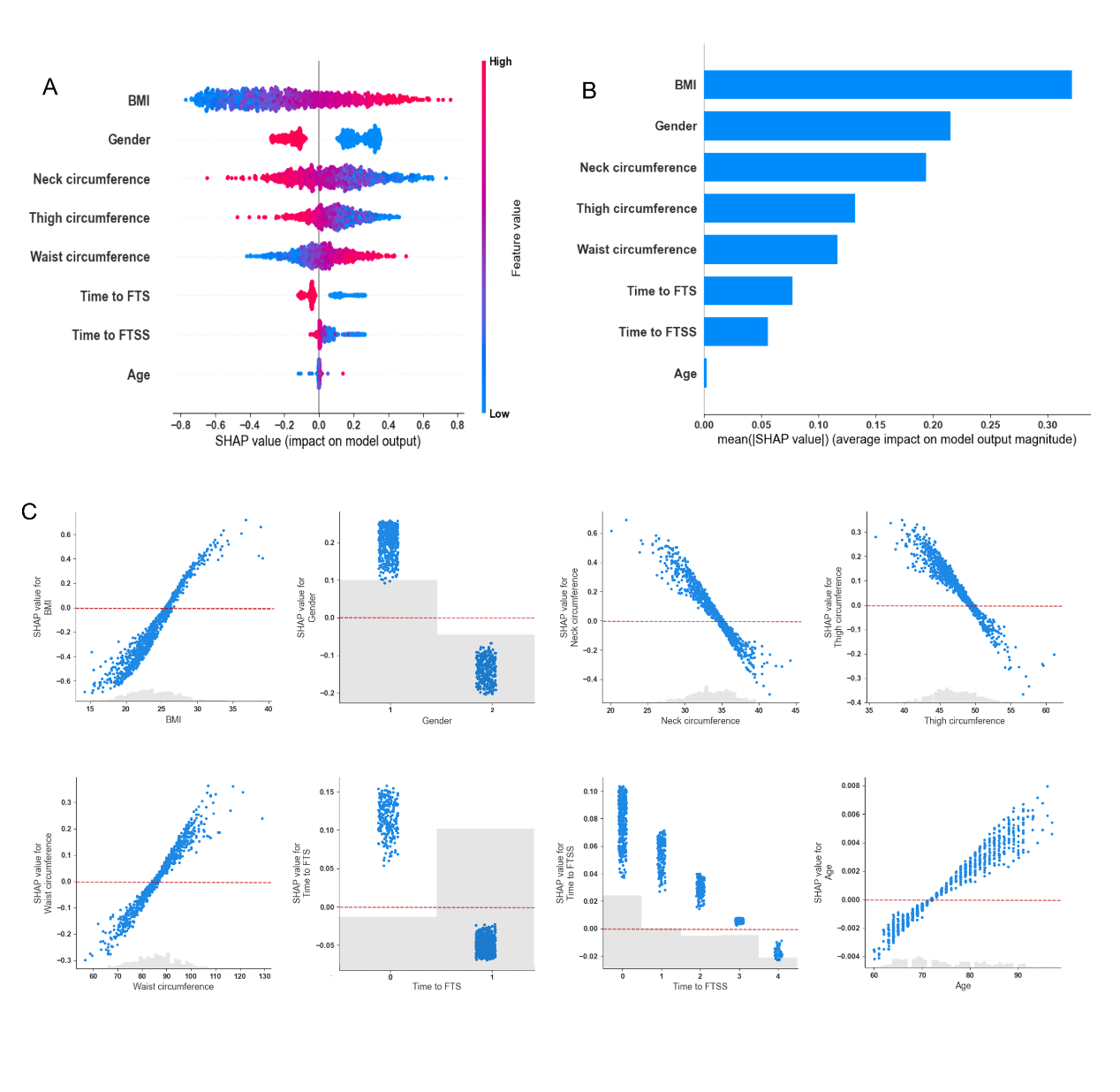


**Figure S4.** Local model explanation by the SHAP method in the external validation set.


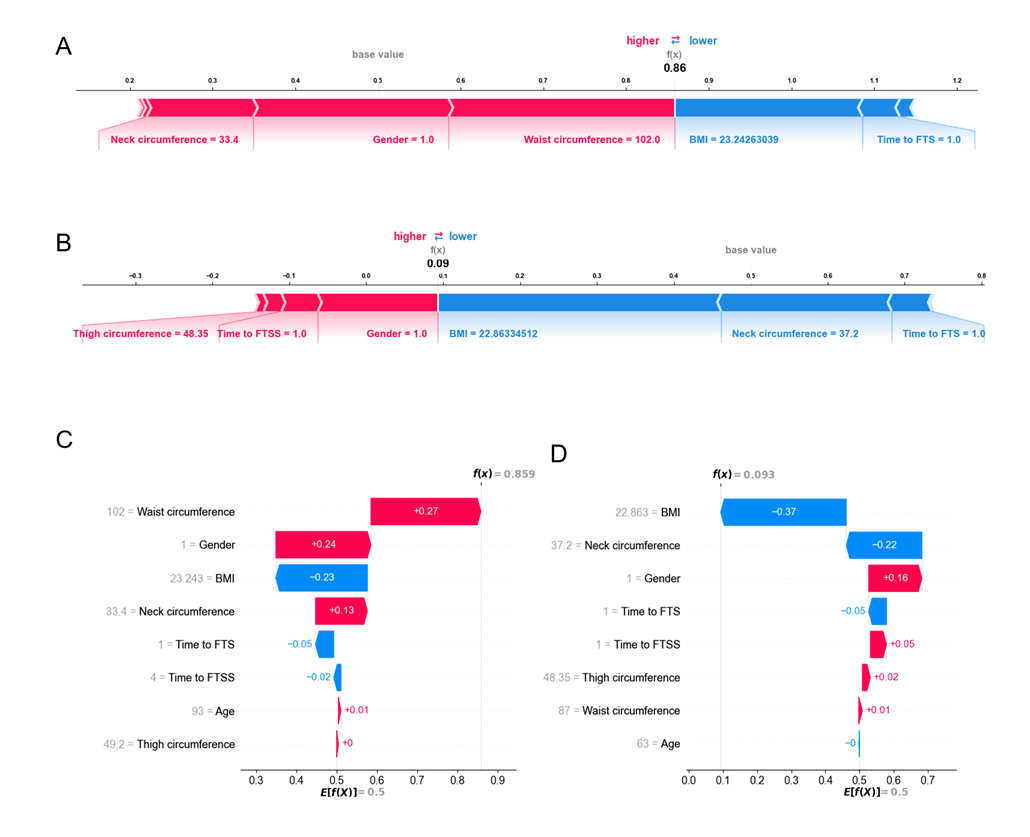

Supplement: Supplementary file 2 — Supplementary Material 2 [file 40520_2025_2975_MOESM2_ESM.docx]
